# Frequency of CD34 and CD10 Expression in Adolescent and Young Adult Patients Having Precursor B-cell Acute Lymphoblastic Leukemia and Its Correlation With Clinical Outcomes: A Single-Center Study

**DOI:** 10.7759/cureus.21261

**Published:** 2022-01-15

**Authors:** Munawar Ali Shah, Usman Ahmad, Muhammad Tariq Mahmood, Asad H Ahmad, Muhammad Abu Bakar

**Affiliations:** 1 Hematology, Shaukat Khanum Memorial Cancer Hospital and Research Centre, Lahore, PAK; 2 Medical Oncology, Shaukat Khanum Memorial Cancer Hospital and Research Centre, Lahore, PAK; 3 Pathology, Shaukat Khanum Memorial Cancer Hospital and Research Centre, Lahore, PAK; 4 Biostatistics and Epidemiology, Shaukat Khanum Memorial Cancer Hospital and Research Centre, Lahore, PAK

**Keywords:** total leukocyte count, platelets count, blast count, disease free survival, overall survival, prognostic factors, cd10, cd34, adolescent and young adults, precursor b cell acute lymphoblastic leukemia

## Abstract

Background: The clinical outcomes of CD34 and CD10 antigens expression in adolescent and young adult (AYA) precursor B-cell acute lymphoblastic leukemia (pre-B-ALL) is not still well established. In the present study, we analyzed the laboratory characteristics and clinical outcomes of 123 AYA pre-B-ALL patients in order to evaluate the possible clinical significance of these markers.

Materials and methods: In the current study clinical data of 123 consecutive AYA pre-B-ALL patients aged 18-39 years old, enrolled in adult hematology-oncology unit from December 2014 to April 2019 was analyzed. Patient clinical outcome was calculated as overall survival and disease-free survival.

Results: Overall, 76.4% of patients showed CD34 expression and CD10 expression was found in 90.2%. CD34 and CD10 expression was associated with higher total leucocyte count, increased peripheral blood blast percentage, and decreased platelet count. Overall survival and disease-free survival were both significantly better in CD34 negative and CD10 negative patients compared to their CD34 positive and CD10 positive counterparts.

Interpretation and conclusion: Expressions of CD34 and CD10 are adverse prognostic factors in AYA pre-B-ALL patients and the presence of these antigens influences the clinical outcome of these patients.

## Introduction

Acute lymphoblastic leukemia is a clonal disorder that is characterized by malignant proliferation and accumulation of immature lymphoid cells (lymphoblast) in the medullary cavity [1]. “B-cell acute lymphoblastic leukemia (B-ALL) is a neoplasm of immature B-cell precursors that typically affects children younger than 6 years but is also encountered in older children and adult populations. The estimated global incidence of B-ALL is around one to five per 100,000 people per year” [2]. Overall about four of every 10 cases of acute lymphoblastic leukemia are in adults. The estimated worldwide annual incidence of adult ALL is about one in 100,000 [3]. In Pakistan, statistical data are not available to know the prevalence and incidence of different malignancies including leukemia, as the tumor registry is not maintained [4]. Immunophenotyping has an important role in the diagnosis of leukemia patients, lineage determination, prognostic subgroups identification, and monitoring of patients after treatment [5]. Cell surface phenotype in lymphoblastic leukemia has variably been found to have prognostic significance [6]. CD10 and CD34 are surface markers that have been reported to have prognostic relevance in childhood lymphoblastic leukemia, but the results were conflicting [7]. CD34 was shown to be expressed in 53% of cases of adult lymphoblastic leukemia and had the worst outcome compared with CD34 negative cases [8]. Positive CD10 expression is associated with favorable clinical outcomes in children [9]. The survival rate of children with positive CD34 and CD10 B-ALL was significantly better than those with negative CD34 and CD10 expression so, CD34 and CD10 expression may have prognostic significance and is associated with favorable clinical outcomes in children [10]. The age of the patients with pediatric ALL ranges from 0 to 14 years, adolescents from 15 to 19 years, young adults from 20 to 39 years, adults from 40 to 60 years; and elderly patients include those beyond the age of 65 years [11]. In this study, we have selected the age group 18-39 years, which comes under adolescents and young adults (AYA). The aim of our study is to determine the significance of CD34 and CD10 expression in AYA pre-B-ALL and their correlation with clinical outcomes. Very few such types of studies have been conducted especially in AYA acute lymphoblastic leukemia.

## Materials and methods

This is a descriptive cross-sectional study done at the pathology department of Shaukat Khanum Memorial Hospital and Research Center Lahore, Pakistan. We retrospectively reviewed the data of 123 consecutive acute pre-B-ALL patients aged 18- 39 years old, enrolled in adult hematology-oncology unit from December 2014 to April 2019. In all the cases, the diagnosis was confirmed by flow cytometry. Flow cytometry was performed on the NAVIOS Flow cytometer (Beckman Coulter, Miami, FL, USA). For flow cytometry whole blood or bone marrow aspirate, samples were used, then added fluorescently conjugated antibodies, and incubated for 15 minutes. After it, the lysing solution was added and incubated for another 10 minutes and then the sample was centrifuged for 5 minutes to separate the supernatant of nucleated cells. This supernatant was discarded and the sample was re-suspended in a fixative solution. For cytoplasmic antibodies, a permeabilizing solution for permeabilizing cell membrane was also added. Then it was run on Navios flow cytometer. Monoclonal antibodies against these antigens CD2, CD4, CD8, 7AAD, CD64, CD3, CD5, CD7, CD10, CD13, CD16, CD19, CD20, CD33, CD34, CD79a, CD11b, CD11c, CD38, CD14, MPO, TdT, Kappa, Lambda, HLA-DR, CD117, Cytoplasmic CD3 and CD45 were used. Antigen density, as interpreted from fluorescence intensity relative to a normal comparative cell type, is expressed as: - (no detectable antigen), + (subnormal antigen density), ++ (normal antigen density), and +++ (increased antigen density). Clinical outcomes were measured by overall survival (OS) and disease-free survival (DFS). OS was defined as the time duration from diagnosis until the date of death or censoring patients alive at the last follow-up date. DFS was defined as the length of time a patient survived without relapse or death from the date of first complete remission or censoring patients alive in continuous complete remission at the last follow-up date. All results were analyzed and calculated using the Statistical Package for the Social Sciences (SPSS) 20.0 software (IBM Corp., NY, USA) and the Microsoft Office Excel software 2007. Frequencies and percentages were calculated for categorical variables whereas means and standard deviations were calculated for continuous variables.

## Results

A total of 123 patients were included, patients had an age range of 18 to 34 years and the mean age at presentation was 22 years. Among those 98(80%) were male and 25(20%) were female. The overall male to female ratio was 4:1. Laboratory characteristics of pre-B-ALL patients at presentation are given in Table 1.

**Table 1:** Laboratory characteristics of 123 precursor B-ALL patients at presentation ALT: alanine transaminase; B-ALL: B-cell acute lymphoblastic leukemia

Variables	Mean	Range
Age (years)	22.2	18-34
Total Leucocyte Count (×10^3^/µL)	13.7	0-252
Peripheral Blasts (%)	39	0-97
Hemoglobin (g/dL)	8.4	3-14
Platelets Count (×10^6^/µL)	76.6	3-725
ALT (U/L)	58.8	3-1195
Serum Albumin (g/dL)	3.5	2-5
Serum Creatinine (mg/dL)	0.64	0.2-3

CD34 expression was found in 94 (76.4%) of 123 patients tested, 27 (23.6%) patients did not express CD34. A comparison of laboratory characteristics of these patients is given in Table 2.

**Table 2:** Comparison of laboratory characteristics of CD34+ and CD34- patients ALT: alanine transaminase

	CD34+ patients (n=94)	CD34- patients (n=27)
Variables	Mean	Mean
Age (years)	22.3	21.5
Total Leucocyte Count (×10^3^/µL)	14.8	9.7
Peripheral Blasts (%)	42	29
Hemoglobin (g/dL)	8.37	8.38
Platelets Count (×10^6^/µL)	70.5	98.6
ALT (U/L)	59.7	55.5
Serum Albumin (g/dL)	3.8	3.6
Serum Creatinine (mg/dL)	0.62	0.72

There was no significant difference between the age of clinical presentation in CD34 positive and CD34 negative patients. The mean of total leucocyte count in CD34 negative patients was low as compared to CD34+ patients. Blasts count in peripheral blood was significantly high (42%) in CD34 positive patients compared to CD34 negative patients where it was 29%. The mean of platelets count was significantly high in CD34 negative patients (98.6×10^6^/µL) to that in CD34 positive patients (70.5×10^6^/µL). ALT, serum albumin, and serum creatinine levels were not significantly different in both groups.

**Figure 1:**
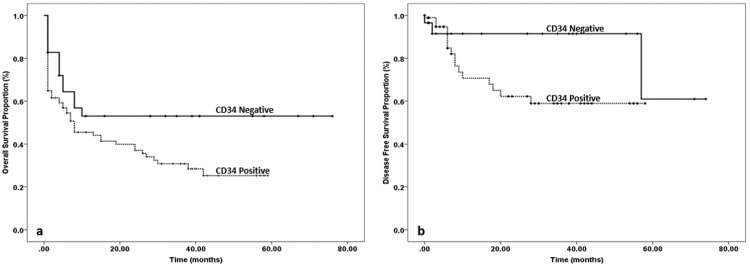
(a) Overall survival (OS) in patients with or without expression of CD34 antigen. (b) Disease-free survival (DSF) in patients with or without expression of CD34 antigen.

OS and DFS in patients with or without expression of CD34 antigen are shown in Figures 1a, 1b.

Five years OS in CD34 negative patients was 53% compared to the survival rate of 25% in CD34 positive individuals. This was statistically significant (log-rank test p-value 0.05). Four years DFS in CD34 negative patients was 91% compared to that of 59% in CD34 positive patients. This was also significantly better in CD34 negative patients.

CD10 expression was found in 111 (90.2%) of patients whereas 12 (9.8%) were CD10 negative patients. A comparison of laboratory characteristics of these patients is given in Table 3.

**Table 3:** Comparison of laboratory characteristics of CD10+ and CD10- patients ALT: alanine transaminase

	CD10+ patients (n=111)	CD10- patients (n=12)
Variables	Mean	Mean
Age (years)	21.9	24.2
Total Leucocyte Count (×10^3^/µL)	13.9	9.9
Peripheral Blasts (%)	39	30
Hemoglobin (g/dL)	8.3	8.5
Platelets Count (×10^6^/µL)	73.4	111.6
ALT (U/L)	60.9	38.1
Serum Albumin (g/dL)	3.7	4.2
Serum Creatinine (mg/dL)	0.64	0.65

The age of initial presentation was slightly low in those who expressed CD10. Total leucocyte count and peripheral blood blast percentage were high in patients with CD10 expression. The mean of platelets counts of CD10+ patients was significantly low as compared to CD10- patients. CD10 positive patients also presented with high ALT and slightly low albumin compared to CD10 negative individuals. There was no difference in creatinine level in both groups.

**Figure 2:**
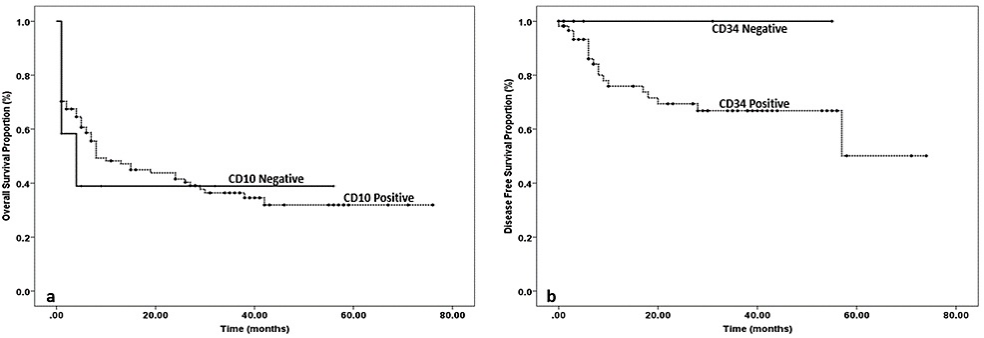
(a): Overall survival (OS) in patients with or without CD10 expression. (b) Disease-free survival (DFS) in patients with or without CD10 expression.

OS and DFS in patients with or without CD10 expression are shown in Figures 2a, 2b.

Five years OS rate was calculated as 40% in CD10 negative patients whereas it was 32% in CD10 positive patients. Thus, OS was slightly better in CD10 negative patients. Five years DFS was 100% in CD10 negative patients whereas it was 50% in CD10 positive patients. It was significantly better in CD10 negative patients compared to their CD10 positive counterparts.

## Discussion

In the current study, 123 AYA Pre-B-ALL patients were included. CD34 was expressed in 76.4% whereas CD10 was positive in 90.2%. A study conducted in Iraq showed positivity of CD34 in 74.7% but that was a mixed population of children and adult B-ALL, in the same study the expression of CD10 was in 83.3% of adult B-ALL [12]. According to Cascavilla et al., the positivity of CD34 in adult B-ALL was 70.5% [13]. The frequency of expression of CD34 and CD10 was almost similar to the previous studies.

 In our study, we noticed that the expression of CD34 and CD10 was associated with adverse markers like higher total leucocyte count, higher peripheral blood blasts percentage, lower platelet count. CD10 positivity was also associated with higher ALT and lower serum albumin levels as well. Similar results were observed by Cascavilla et al. stating that adult CD34+ ALL were prevalently associated with adverse parameters [13]. In another study, 75 newly diagnosed adult ALL patients were analyzed for CD34 positivity. CD34 expression was associated with features of poor prognosis [14].

 Regarding CD10 expression, a study conducted in China showed CD10 negativity in 11.5% of adult pre-B-ALL patients whereas in our study it was 9.8%. Six adult cases of pre-B-ALL patients were analyzed in this study and showed a high mean WBC count (101.78×10^3^/µL) and MLL-AF4 fusion transcript in all [15]. This study conflicted with our results where CD10 negativity was associated with favorable parameters at presentation. German multicenter trials for adult ALL (GMALL) also showed that CD10 negative pre-B ALL is a distinct high-risk subgroup of adult ALL associated with a high frequency of MLL aberrations.

According to our study, the clinical outcome was significantly better, in terms of both OS and DFS, in both CD34 negative and CD10 negative patients compared to CD34 positive and CD10 positive patients respectively. According to a study conducted by Thomas et al. adult ALL CD34 expression was associated with major adverse prognostic factors, CD34 positivity was associated with the persistence of blasts in day 15 bone marrow aspirate and after one course of induction chemotherapy, however, no significant statistical differences were seen in DFS and OS between CD34 positive and CD34 negative cases [14]. Another study by Cascavella et al. also showed similar results to Thomas et al. that CD34 was expressed in poor-risk B-ALL patients and associated with features of poor prognosis [13]. Our study confirmed the results of these studies and extended it as there was a significant statistical difference in OS and DFS between CD34 positive and CD34 negative patients.

The study by Gleissner et al. showed CD10 negative pre-B ALL was a distinct high-risk group of adult ALL, especially those with MLL gene rearrangements were having worse outcomes [16]. The other study conducted by XY Gongnet et al. also revealed a similar type of results with CD10 negative cases [15]. Our study opposed both of these studies and showed that expression of CD10 was associated with adverse prognostic markers and had inferior clinical outcomes compared to CD10 negative cases.

A retrospective study design and relatively small study size account for the limitation of our study. Similarly, the data had been collected from one institution which limits the generalizability of our results. Co-expression of CD34 and CD10 in their outcomes had not been studied.

## Conclusions

We concluded that expression of D34 and expression of CD10 were frequent events in AYA pre-B-ALL. In this study, their expression was associated with poor prognostic features and clinical outcomes. Detection of CD34 or CD10 on blast cells had a possibly independent negative prognostic impact in AYA pre-B-ALL. Further studies are needed to evaluate it more in AYA pre-B-ALL population.
